# Dual Inhibition of P-gp and BCRP Improves Oral Topotecan Bioavailability in Rodents

**DOI:** 10.3390/pharmaceutics13040559

**Published:** 2021-04-15

**Authors:** Jaeok Lee, Jiyeon Kang, Na-Yun Kwon, Aneesh Sivaraman, Ravi Naik, So-Young Jin, A. Reum Oh, Jae-Ho Shin, Younghwa Na, Kyeong Lee, Hwa-Jeong Lee

**Affiliations:** 1 College of Pharmacy and Graduate School of Pharmaceutical Sciences, Ewha Womans University, Seoul 03760, Korea; leejo19@ewha.ac.kr (J.L.); kkangji@ewhain.net (J.K.); kny5754@naver.com (N.-Y.K.); heavenward_@naver.com (S.-Y.J.); dhdkf9922@ewhain.net (A.R.O.); 2 College of Pharmacy, Dongguk University, Goyang-si 10326, Korea; aneeshdongguk@gmail.com (A.S.); Mravi.naik@gmail.com (R.N.); kaylee@dongguk.edu (K.L.); 3 College of Pharmacy, CHA University, Pocheon-si 11160, Korea; wogh975@naver.com (J.-H.S.); yna7315@chauniv.ac.kr (Y.N.)

**Keywords:** P-gp and BCRP dual inhibition, topotecan, excipient, oral bioavailability, pharmacokinetics, tumor growth

## Abstract

P-glycoprotein (P-gp) inhibition has been studied to overcome multidrug resistance in cancer chemotherapy but failed in clinical trials due to low/toxic effects. Recently, a dual modulation of transporters and natural derivatives have been examined to surmount this limitation. We examined breast cancer resistance protein (BCRP) inhibition in vitro and in vivo by P-gp inhibitors derived from natural compounds in previous studies. P-gp inhibitors increased the accumulation of the anticancer drug, topotecan (TPT)—a substrate of P-gp and BCRP, albeit with higher affinity for BCRP—in BCRP-overexpressing cells, resulting in cell death. These dual inhibitors, when orally co-administered with TPT, enhanced TPT bioavailability with slightly reduced total oral clearance (Clt/F) in rats. In xenograft mice, they strengthened oral TPT-induced tumor reduction with no alterations in body weight. Moreover, we investigated the effects of an oral drug formulation (Cremophor^®^ EL, Tween^®^ 80, and polyethylene glycol 400) on the transporters function. The excipients increased TPT accumulation in P-gp- or BCRP-overexpressing cells. Oral TPT bioavailability was higher with the formulation than with a control, as shown by the increases in the maximum plasma concentration (C_max_) and the area under the plasma concentration–time curve from zero to infinity (AUC_INF_) (*p* *<* 0.01). Therefore, oral TPT bioavailability was enhanced by P-gp/BCRP dual inhibition, which resulted in a formulation-mediated increase in absorption and decrease in elimination, and a dual inhibitor-mediated decrease in elimination. These results suggest that the combination of dual inhibition by a natural derivative and the drug formulation can be a useful clinical approach.

## 1. Introduction

Adenosine triphosphate (ATP)-binding cassette (ABC) transporters belong to one of the largest transporter families and act as active ATP-dependent efflux pumps in living organisms [1,2]. The function of these ABC transporters is intimately associated with multidrug resistance (MDR) in cancer treatment [3]. P-glycoprotein (P-gp)—ABC subfamily B member 1 (ABCB1)—is a well-characterized MDR transporter, and was the first transporter to be identified [4]. Breast cancer resistance protein (BCRP)—ABCG2—is also a crucial MDR transporter and is a recently identified and less-characterized transporter among the ABC transporters [5]. P-gp and BCRP are related not only to pathogenic conditions, but also to normal physiological roles. They are widely expressed in important tissues such as the intestinal epithelium, the biliary canaliculi of hepatocytes, the proximal tubules of kidney, and the endothelium at the blood–brain barrier (BBB) [6]. Although both transporters have different sizes, structures, and domain arrangement [3,7], many anticancer drugs have been discovered from their common substrates [8,9].

In addition, ABC transporters strongly influence the absorption, distribution, metabolism, and excretion of drugs [10]. Therefore, the United States Food and Drug Administration (US-FDA) has formulated guidelines for the evaluation of P-gp- and BCRP-mediated drug interactions [11]. Because efflux transporters are the one of the primary causes of MDR and restricted BBB penetration, and can alter the pharmacokinetic (PK) properties of various anticancer drugs, their modulation has been of interest in addressing MDR, BBB penetration, and improving the oral bioavailability (BA) of substrate drugs [12,13]. Most studies of transporter modulation have focused on single transporters [14,15]. Numerous studies have investigated P-gp modulation by molecules beyond the third generation of inhibitors, which have a high specificity and a low risk of adverse reactions, but show a lack of significant effects or unexpected toxicity in clinical trials [14,16]. Recently, a fourth generation of P-gp inhibitors using natural compounds or their derivatives and P-gp/BCRP dual-target modulation have been proposed for clinical investigation [16,17,18].

Meanwhile, many studies have focused on the interaction of excipients, such as surfactants present in oral drug formulations, with P-gp and BCRP [19,20]. Cremophor^®^ EL, Tween^®^ 80, and polyethylene glycol (PEG) 400 are typical nonionic surfactants and excipients whose effects have been investigated on P-gp or BCRP modulation [19]. Cremophor^®^ EL increased the accumulation of P-gp or BCRP substrate drugs, such as daunorubicin (DNR) and mitoxantrone (MX), and Tween^®^ 80 and PEG 400 also enhanced the accumulation of P-gp substrate drugs (Rh123, epirubicin) in different cell lines such as R100, Caco-2, and MDCK1 [19]. According to these reports, the proper use of excipients in the formulation of P-gp and BCRP substrate anticancer drugs would be a potent strategy for the enhancement of oral BA. However, preclinical evidence for the effects of excipients on P-gp or BCRP modulation is still limited because most evidence is based on in vitro studies. Moreover, the effect on P-gp and BCRP dual modulation has not been studied yet to our knowledge.

Topotecan (TPT), a water-soluble camptothecin analog that inhibits the catalytic activity of DNA topoisomerase I, is a widely used anticancer drug against various cancers, including ovarian, lung, and other cancers [21]. TPT is administered through the intravenous (IV) and per oral (PO) routes [21]. Under physiological pH, TPT is unstable because its lactone moiety is converted to a carboxylated open-ring form, which has low topoisomerase I inhibitory activity [22]. In humans, the oral BA of active TPT is reported to be approximately 35%. It is eliminated by both renal excretion (approximately 40% of the total administered dose) and hepatic metabolism (low amount of *N*-desmethyl TPT (<7% of the total amount), which is most likely metabolized by cytochrome P450 3A (CYP3A)) with a half-life of 3 h [22]. This anticancer drug is classified as a substrate of P-gp and BCRP transporters, with a higher affinity for BCRP [8,9,23]. Therefore, the inhibition of both transporters during TPT treatment has been proposed to enhance oral BA and BBB penetration, as well as for reversing MDR. Few studies have investigated this dual inhibition [23,24,25,26].

Previously, our studies have investigated P-gp modulation with numerous derivatives of natural compounds, including coumarin derivatives, optically active phenylbutenoid dimers, xanthone analogues, benzoxanthone analogues, piperazine derivatives, and ferulic acid derivatives [27,28,29,30,31,32]. Here, we assessed P-gp and BCRP dual-modulatory activity with some P-gp modulators from our previously reported series of natural compound derivatives [27,29,30] to confirm the possibility that P-gp modulators could be developed as P-gp/BCRP dual-target modulators—another concept of fourth generation modulation. Among the selected compounds, compounds **2** [30], **LL344** [27], and **13-1** (modified from xanthone analogue **13** [29]) (Figure S1), increased the oral BA of TPT, a substrate of both transporters, in rats, and enhanced tumor growth reduction in human colon cancer cell xenograft-bearing nude mice when co-administered orally with TPT. Moreover, we demonstrated that the modified Taxol^®^ formulation containing Cremophor^®^ EL, Tween^®^ 80, and PEG 400 had dual modulatory activity by improving oral TPT absorption in these animals.

## 2. Materials and Methods

### 2.1. Materials

P-gp and BCRP dual inhibitor candidates (approximately 99% purity) were synthesized by K. Lee [27] and Y. Na [30]. TPT, mitoxantrone (MX), and DNR were purchased from Mesochem (Beijing Mesochem Technology Co., Ltd., Beijing, China). Zosuquidar (MedChemExpress, Monmouth Junction, NJ, USA), Ko143 (Abcam, Cambridge, UK), radioimmunoprecipitation assay (RIPA) buffer (CellNest, Tokoy, Japan), protease inhibitor (Calbiochem, San Diego, CA, USA), and antibodies against P-gp (C219, Enzo life sciences AG., Lausen, Switzerland), BCRP (BXP21, Santa Cruz Biotechnology, Inc., CA, USA), β-actin (C4, Santa Cruz Biotechnology, Inc., CA, USA), and goat anti-mouse IgG horseradish peroxidase-conjugated antibody (EMD Millipore, Billerica, MA, USA) were commercially obtained. Cremophor^®^ EL, Tween^®^ 80, PEG 400, dimethylsulfoxide (DMSO), and acridine were purchased from Sigma-Aldrich (Millipore Sigma Co., Burlington, MA, USA). Zoletil 50 (Virbac S.A., Carros, France, USA), Rumpun^®^ injection (Bayer Korea, Seoul, Korea), isoflurane (Hana Pharm Co., Ltd., Seoul, Korea), heparin sodium injections (Joongwae Pharm., Seoul, Korea), and Matrigel^®^ (Corning^®^, Corning, NY, USA) were purchased. All reagents and solvents were of molecular biology and cell culture grades, animal experiment grade, or high-performance liquid chromatography (HPLC) analysis grade.

The human breast cancer cell lines MCF-7, MCF-7/ADR (P-gp-overexpressing), and MCF-7/MX100 (BCRP-overexpressing) were kindly gifted by Marilyn E. Morris (University of Buffalo, NY, USA), and the human colorectal cancer cell line, HT29, was presented by U. Schumacher (University Medical Center of Hamburg-Eppendorf, Hamburg, Germany). Laboratory animals, Sprague–Dawley (SD) rats and Balb/c nude mice, were purchased from Orient Bio (Seongnam, Korea).

### 2.2. Cell Culture

Parental, MCF-7/ADR, and MCF-7/MX100 cells were maintained in Dulbecco’s Modified Eagle Medium (DMEM) media, DMEM containing 1 μM doxorubicin, and DMEM containing 0.1 μM MX, respectively, and HT29 cells were cultured in Roswell Park Memorial Institute (RPMI) 1640 media at 37 °C in a humidified 5% CO_2_ atmosphere. DMEM media were supplemented with 25 mM D-glucose, 4 mM L-glutamine, 1 mM sodium pyruvate, 44 mM NaHCO_3_, and 10% fetal bovine serum, and RPMI 1640 media were supplemented with 2 mM L-glutamine, 0.1 mM L-methionine, 24 mM NaHCO_3_, and 10% fetal bovine serum.

### 2.3. Western Blotting Analysis

MCF-7/ADR and MCF-7/MX100 cells incubated with P-gp and BCRP dual inhibitor candidates for 48 h were lysed and sonicated in RIPA buffer containing a protease inhibitor cocktail. Fifty micrograms of each protein sample were electrophoretically separated in a 7.5% sodium dodecyl sulfate-polyacrylamide gel electrophoresis (SDS-PAGE) gel under reducing conditions, followed by transfer to an activated polyvinyldifluoride membrane. After blocking with 5% skimmed milk solution, the membrane was incubated overnight with primary antibodies against P-gp (1:250), BCRP (1:100), and β-actin (1:2000) at 4 ℃. This was followed by incubation with the secondary antibody (1:2000‒1:10,000). The horseradish peroxidase reaction for the detection of target protein expression levels was monitored using a ChemiDOC MP (Universal Hood III #731BR02006, Bio-Rad Laboratory, USA), and target protein levels were measured with ImageJ software (version 1.52n, NIH, Bethesda, MD, USA). Three independent experiments were analyzed.

### 2.4. Cell Survival Study

MCF-7/ADR and MCF-7/MX100 cells (9 × 10^3^/well) in 96-well plates were incubated with TPT with or without dual inhibitor candidates for 48 h. Cell survival was examined using the sulforhodamine B staining assay [33]. Absorbance was measured at 515 nm with a spectrophotometer (Multiskan GO with Cuvette function #1510-04234, Thermo Fisher Scientific, Vartaa, Finland). The assay was performed at least in triplicate.

### 2.5. Substrate Drug Accumulation Study by Flow Cytometry

The trypsinized cells (1 × 10^6^‒3 × 10^6^) were incubated in prewarmed phenol red-free media (500 μL) with 200 μM TPT with or without zosuquidar, Ko143, each dual inhibitor candidate, Cremophor^®^ EL, Tween^®^ 80, and PEG 400 for 30 min, or with 10 μM DNR in the presence and absence of compound **13-1** for 30 min at 37 ℃, after washing with media through a 26 G syringe needle. In this study, the cells were treated with TPT or DNR after incubation with the P-gp and/or BCRP inhibitor candidates or with the excipients for 5 min. When the incubation was complete, the medium containing the substrate and inhibitor candidate or excipient was removed, and the cells were resuspended in cold phosphate buffer saline (PBS) (500 μL). TPT accumulation in the cells was measured using a flow cytometer (NovoCyte 2060R, ACEA Biosciences Inc., San Diego, CA, USA). The mean fluorescence intensity values were calculated, and at least three independent biological duplicates were performed.

### 2.6. Animal Experiments

All animal experiments were performed as per the procedures approved by the Ewha Womans University Institutional Animal Care and Use Committee (IACUC), Korea (No. 2019-19-002 approved on 11 January 2019 for the pharmacokinetic study, No. 2016-16-062 approved on 7 December 2016, and No. 2018-18-072 approved on 29 December 2018 for the xenograft study).

#### 2.6.1. Pharmacokinetic (PK) Studies

##### Drug Formulations

TPT at 2 mg/mL was prepared immediately prior to use using the modified Taxol^®^ formulation (Cremophor^®^ EL, PEG 400, Tween^®^ 80, and an isotonic saline solution (25/5/0.5/69.5, *v*/*v/v*/*v*) mixed with DMSO (9:1, *v*/*v*)). Compounds **2**, **3**, **LL344**, **LL347**, and **13-1** (Figure S1) were dissolved in DMSO at concentrations of 2 mg/mL for PK studies in rats and 4 mg/mL for xenograft studies in mice. Each compound dissolved in DMSO was mixed with TPT in the modified Taxol^®^ formulation without DMSO for oral administration to rats and mice.

##### Oral Administration and Plasma Sampling

For PK studies, the carotid artery in healthy SD rats (male, 7‒8 weeks, 220‒298 g) was catheterized one day before drug administration [29,30,31,32]. Four to six rats were grouped for the oral administration of TPT (20 mg/kg) with or without compounds (2 mg/kg) in the modified Taxol^®^ formulation and the same dose of TPT in saline only. After drug administration, blood (approximately 250 μL) was sampled from the catheter at 0, 0.05, 0.12, 0.25, 0.5, 1, 2, 3, 4, 6, 10, and 24 h, and an equal volume of heparinized saline was refilled through the catheter to maintain total blood volume and prevent blood clotting. Plasma samples separated by centrifugation of blood at 13,000 rpm for 15 min at 4 ℃ were stored at −20 ℃ or −70 ℃ until analysis.

##### High Performance Liquid Chromatography-Fluorescence (HPLC-FC) Analysis

TPT concentrations in rat plasma were analyzed using an Agilent 1260 infinity system (Agilent Technology Inc., Waldbronn, Germany) equipped with Agilent ChemStation software (version Rev.B.04.03-SP2 (105), Agilent, Santa Clara, CA, USA), a quaternary pump, a degasser, a thermostatic autosampler, a thermostatic column compartment, and a fluorescence detector. The samples were chromatographically separated using a Capcell Pak C_18_ UG120 column (4.6 mm × 250 mm, 5 μm, Osaka Soda Co. Ltd., Osaka, Japan) with the mobile phase composed of 10 mM phosphate buffer containing 1% triethylamine (TEA) (pH 3.5) and methanol containing 1% TEA (pH 6.5) (56/44, *v*/*v*) at a flow rate of 1.0 mL/min for 30 min and detected at an excitation wavelength of 381 nm and an emission wavelength of 527 nm. The injection volume was 50 μL. The HPLC-FC method was validated according to US-FDA guidelines [34] as follows: specificity (TPT: 3.8 min; internal standard (IS): 18.1 min), linearity over the concentration range between 0.025 and 5.0 μg/mL (*y* = 0.2679*x* + 0.0013, coefficient of determination (r^2^) = 0.9997), lower limit of quantification (0.025 μg/mL), intra-/inter-day precision and accuracy (3.7–12.2% and 91.3–112.6%, respectively), and the short term, long term, freeze-thaw, and post-preparation stability of TPT (between 87.0% and 102.0%), and extraction recovery of TPT and IS (between 91.21% and 94.24%).

For the HPLC-FC analysis, TPT samples in rat plasma were prepared as follows: 33 μL plasma was mixed with 3 μL acridine (IS) (22.2 μg/mL), and the mixture was deproteinized with 84 μL cold methanol and 30 μL 100 mM phosphoric acid. The plasma sample was ready to inject after vertexing for 2 min, and then centrifuging at 13,000 rpm for 15 min.

##### PK Analysis

Pheonix WinNonlin^®^ software (version 8.1, Pharsight Corporation, Mountain View, CA, USA) was used to estimate the PK parameters following oral TPT administration to rats. Non-compartmental analysis was performed using the plasma TPT concentration–time profiles to obtain the following PK parameters: area under the plasma concentration–time curve from zero to infinity (AUC_INF_), elimination half-life (t_1/2_), apparent volume of distribution after oral administration (V_d_/F), and total oral clearance (Cl_t_/F). The maximum plasma concentration (C_max_) and the time required to reach C_max_ (T_max_) were directly measured from the plasma TPT concentration–time curve. The relative BA (RB) of TPT was calculated using the Equation (1):

RB (%) = (AUC_INF_ po co-administration)/(AUC_INF_ po control) × 100 (1)

#### 2.6.2. Xenograft Trial

HT29 cells (3.5 × 10^6^ in 50 µL Dulbecco’s phosphate-buffered saline–Matrigel^®^ mixture (1:1)), expressing both P-gp and BCRP proteins endogenously, were subcutaneously inoculated into the flanks of Balb/c nude mice (male, 5-week, 18‒20 g). TPT (1 mg/kg) with or without inhibitor candidates (2 mg/kg), and the combination of zosuquidar and Ko143 (2 mg/kg each) versus the control (the modified Taxol^®^ formulation itself) were orally administered twice weekly for 3‒3.5 weeks when the tumor volume reached approximately 100 mm^3^ (*n* = 5‒7/group). In this study, the inhibitor candidates and the positive control were administered 30 min before TPT treatment. The mouse tumor volume was measured with digital calipers, and body weight was monitored twice a week. The mice were euthanized when the tumor volume reached 2000 mm^3^ or in case of any signs of distress.

### 2.7. Statistical Analysis

Tukey’s honestly significant difference or Dunnett’s T3 test in conjunction with one-way ANOVA (Free GraphPad Prism, version 8.3.0, La Jolla, CA, USA) was used for the statistical analysis of Western blotting data, cell survival data, TPT accumulation data, and PK analyses of TPT with the inhibitor candidates. Student’s *t*-test/F-test, repeated-measures one-way ANOVA, and the Mann–Whitney U test (GraphPad Prism, version 8.3.0) were performed for the analysis of the effects on DNR accumulation of compound 13-1 and on tumor xenograft growth, and the PK of TPT in saline vs. the modified Taxol^®^ formulation, respectively. PK parameters were represented as mean ± standard deviation (S.D.), and other data were represented as mean ± standard error of the mean (S.E.M.). Statistical significance was represented by *p* values < 0.05.

## 3. Results

### 3.1. Effects of P-gp Inhibitors on BCRP In Vitro

**Figure 1:**
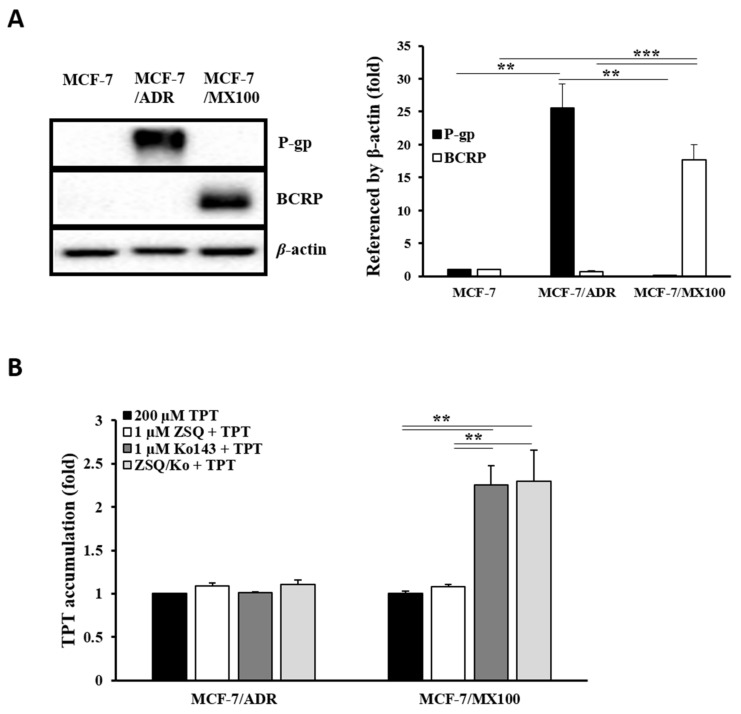
P-gp- and BCRP-overexpressing in vitro system. (**A**) Western blotting analysis using anti-P-gp and anti-BCRP antibodies in ADR and MX100 cell lines versus the MCF-7 cell line. (**B**) TPT (200 μM) accumulation after co-treatment with zosuquidar (1 μM), Ko143 (1 μM), and the combination of both (1 μM each) in ADR and MX100 cells. P-gp: P-glycoprotein; BCRP: breast cancer resistance protein; TPT: topotecan; ZSQ: zosuquidar; ZSQ/Ko: the combination of zosuquidar and Ko143. ** *p* < 0.01; *** *p* < 0.001.

To determine whether the previously reported P-gp inhibitors could inhibit BCRP, we examined the effects of specific inhibitors on P-gp and BCRP function by monitoring TPT accumulation in P-gp- and BCRP-overexpressing cells (Figure 1). Compared to the parental cells, MCF-7/ADR and MCF-7/MX100 cells showed approximately 25-fold and 18-fold higher expression of P-gp and BCRP, respectively (Figure 1A). We observed over two-fold accumulation of TPT in MX100 cells (*p* < 0.01) when BCRP function was inhibited by its specific inhibitor (Ko143) compared with TPT-only treatment (Figure 1B). However, no significant difference was observed in TPT accumulation in ADR cells treated with a P-gp specific inhibitor (zosuquidar). P-gp function in ADR cells was confirmed with the accumulation of daunorubicin (DNR), a well-known P-gp substrate (Figure S2A). The P-gp specific inhibitor, zosuquidar, induced an approximately three-fold increase in DNR accumulation (versus 10 μM DNR-only control, *p* < 0.05). However, no significance difference was observed in TPT accumulation in the case of the combination treatment with P-gp- and BCRP-specific inhibitors compared to that observed separately for the inhibition of each transporter in both cell lines. Thus, these results demonstrate the suitability of this in vitro system for the subsequent experiments and confirm the higher affinity of TPT for BCRP in the present in vitro system.

**Figure 2:**
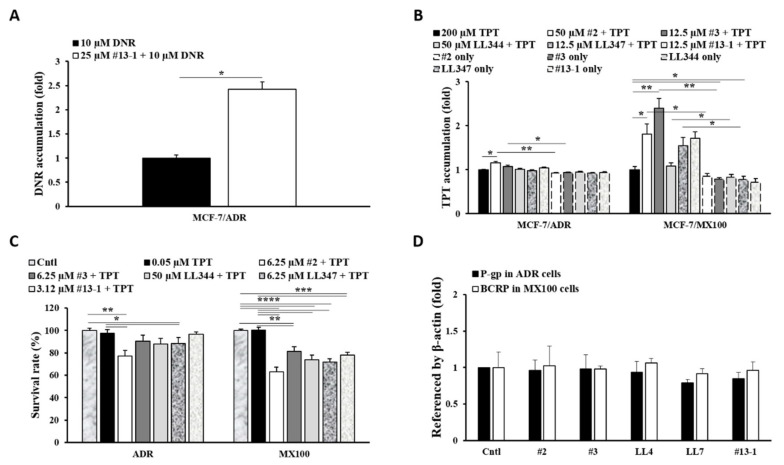
Decreased TPT efflux by P-gp inhibitors. (**A**) DNR accumulation after co-treatment with compound **13-1** in ADR cells. (**B**) TPT accumulation after co-treatment with compounds **2**, **3**, **LL344**, **LL347**, and **13-1** in ADR and MX100 cells. (**C**) Cell survival analysis after TPT co-treatment with the compounds (48 h) in ADR and MX100 cells. (**D**) Western blotting analysis of P-gp and BCRP protein expression levels during long-term treatment with compounds (48 h). Cntl: control; **#2**: compound 2; **#3**: compound 3; **LL344**: compound LL344; **LL347**: compound LL347; **#13-1**: compound 13-1; DNR: daunorubicin. * *p* < 0.05; ** *p* < 0.01; *** *p* < 0.001; **** *p* < 0.0001.

The P-gp inhibitors, compounds **2**, **3** [30], **LL344**, **LL347** [27], and **13-1** (modified xanthone analog **13** [29]) were examined for their potential as BCRP inhibitors in the in vitro system described above (Figure 2). Owing to its effect on DNR accumulation, we confirmed the inhibitory activity of compound **13-1** toward P-gp efflux (Figure 2A). The compound increased DNR accumulation by approximately 2.5-fold in P-gp-overexpressing cells compared to the DNR-only control (*p* < 0.05). All compounds, confirmed to be P-gp inhibitors in vitro, were investigated for the inhibition of BCRP-mediated efflux activity through the analysis of TPT accumulation (Figure 2B). Compounds **2** and **3** significantly induced drug accumulation in MX100 cells (*p* < 0.05 and *p* < 0.01, respectively) to levels that were comparable to that observed for the specific BCRP inhibitor (Ko143) (Figure 1B). Compounds **LL347** and **13-1** showed an increase in TPT accumulation in BCRP-overexpressing cells. Moreover, compound **2** significantly increased TPT accumulation in P-gp-overexpressing cells (*p* < 0.05). Because TPT accumulation in the cells after short-term incubation (up to 2 h) was much lower than that observed for other drugs (Figure S2B), the duration of incubation was increased to 48 h (Figure 2C). All compounds significantly increased TPT cytotoxicity versus the TPT-only control in BCRP-overexpressing cells (*p* < 0.01–0.0001) after co-treatment with 0.05 μM TPT, a non-toxic concentration of TPT. Increased TPT cytotoxicity was observed in P-gp-overexpressing cells after treatment with the P-gp inhibitors, except for compound **13-1,** with significant cytotoxicity being observed for compounds **2** and **LL347** versus the TPT-only control (*p* < 0.05). However, there were no significant changes in the protein levels of both efflux transporters after treatment with the compounds (Figure 2D).

### 3.2. Effects of Dual Inhibitor Candidates In Vivo: Pharmacokinetics (PK) of TPT Following Oral Co-Administration

**Figure 3:**
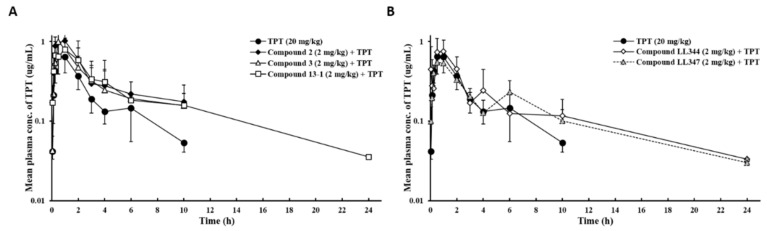
Mean plasma concentration–time profiles following the oral co-administration of TPT with P-gp and BCRP dual inhibitor candidates to rats. (**A**) Benzoxanthone and xanthone derivatives and (**B**) coumarin derivatives. Bars represent S.D. (*n* = 4–6). TPT (20 mg/kg, PO) was orally administered alone or with each compound (2 mg/kg) to rats. S.D., standard deviation; PO, per oral.

Compounds whose dual inhibition of P-gp and BCRP had been confirmed in vitro were investigated for their inhibitory effects in our animal model. TPT (20 mg/kg) dissolved in the modified Taxol^®^ formulation containing Cremophor^®^ EL, PEG 400, Tween^®^ 80, and isotonic saline solution mixed with DMSO (9:1, *v*/*v*) was orally administered to rats with or without each compound (2 mg/kg). Figure 3 and Table 1 show the mean plasma concentration–time profiles and PK parameters of TPT, respectively. In spite of the lack of statistical significance, all the compounds increased the AUC_INF_ of TPT by approximately 1.4- to 2.0-fold, with a moderate decrease in Cl_t_/F. Compound **2** exhibited the most prominent effects in the in vitro study (Figure 2B,C), where it demonstrated 1.6- and 2.1-fold increases in the C_max_ and RB of TPT, respectively, whereas compound **13-1** demonstrated approximately 2.0-fold increase in the RB of TPT compared to TPT only (Figure 3 and Table 1). In addition, the T_max_ of TPT was similar in the presence or absence of the compounds, and the second peak was monitored in the plasma TPT concentration–time profile after co-administration with compounds **LL344** (4 h) and **LL347** (6 h) (Figure 3).

### 3.3. Effects of Dual Inhibitor Candidates In Vivo: Tumor Growth after Oral Co-Administration with TPT

**Figure 4:**
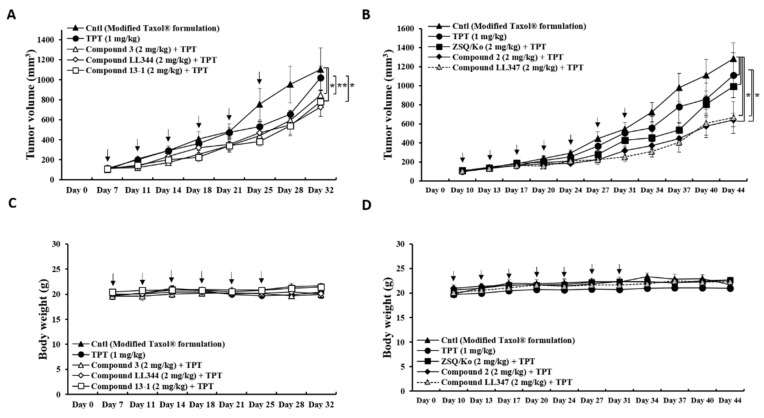
Reduction in tumor growth by oral TPT co-administration with the dual inhibitor candidates. (**A**) Change in tumor volume after co-treatment of TPT with compounds **3**, **LL344**, and **13-1** versus TPT-only and vehicle-only controls twice a week (*n* = 5–7). (**B**) Change in tumor volume after co-treatment of TPT with compounds **2** and **LL347** versus TPT-only, vehicle-only, and positive controls (co-treatment with the combination of zosuquidar and Ko143) twice a week (*n* = 5). (**C**) Change in body weight after treatment scheme described in A. (**D**) Change in body weight after treatment scheme described in B. Bars represent S.E.M. Cntl: vehicle-treated control; TPT: 1 mg/kg; each compound: 2 mg/kg; ZSQ/Ko: the mixture of zosuquidar and Ko143, 2 mg/kg each. * *p* < 0.05; ** *p* < 0.01.

To evaluate the effects of the dual inhibitors on tumor growth, each compound (2 mg/kg) was orally co-administered with TPT (1 mg/kg) to tumor xenograft bearing mice, and the tumor volume and body weight of the mice were monitored on predetermined days (Figure 4). We generated the tumor xenograft model with human colon cancer cells (HT29) which endogenously express both P-gp and BCRP proteins [35] (Figure S3A), as P-gp and BCRP are expressed in the small intestine and colon [36]. The effects of orally administering the low dose of the dual inhibitor candidates (2 mg/kg) with the low dose of TPT (1 mg/kg) on tumor growth was investigated after confirming TPT sensitivity in HT29 cells (Figure S3B). At the end point, all compounds co-administered with TPT (multiple dosing) showed markedly smaller tumor volumes than the TPT-only group and the vehicle-treated control group (Figure 4A,B). Except for compound **LL344,** all the dual inhibitors significantly induced tumor reduction by TPT, compared to TPT only and the formulation only control (control vs. compound **2**, *p* < 0.05; control vs. compound **3**, *p* < 0.05; control vs. compound **LL347**, *p* < 0.05; control vs. compound **13-1**, *p* < 0.05; TPT vs. compound **2**, *p* < 0.05; TPT vs. compound **3**, *p* < 0.01; TPT vs. compound **LL347**, *p* < 0.05; TPT vs. compound **13-1**, *p* < 0.05). The tumor reduction value induced by compound **LL344**, however, was not statistically different from other compounds (Figure 4A). Moreover, the reduction induced by dual inhibitors was more enhanced than by the zosuquidar and Ko143 mixture, although there was no statistical difference (Figure 4B). In addition, the experimental mice did not show any notable change in their body weight during the treatment period (Figure 4C,D).

### 3.4. Effects of Excipients on P-gp and BCRP Dual Inhibition In Vitro and In Vivo

**Figure 5:**
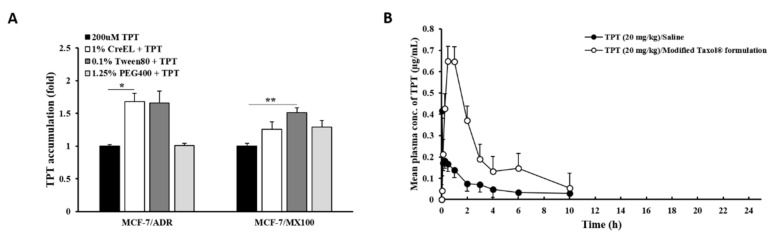
Effects of excipients on TPT accumulation and absorption. (**A**) TPT accumulation in the presence and absence of each excipient in P-gp- or BCRP-overexpressing cell lines. (**B**) Mean plasma concentration–time profiles of TPT (20 mg/kg) following the oral administration of different formulations of TPT to rats. Bars represent S.D. (*n* = 6-7). * *p* < 0.05; ** *p* < 0.01.

We investigated the effects of the modified Taxol^®^ formulation used in the present study on TPT absorption after P-gp and BCRP inhibition (Figure 5). The effect of each excipient—Cremophor^®^ EL, Tween^®^ 80, and PEG 400— in the formulation on TPT accumulation was monitored in vitro (Figure 5A). The working concentration of each excipient—1.00%, 0.10%, and 1.25%, respectively— caused no significant cytotoxicity (over 90% survival rate). Cremophor^®^ EL and Tween^®^ 80 considerably increased TPT accumulation compared to the TPT control in P-gp-overexpressing cells, while all excipients slightly induced TPT accumulation in BCRP-overexpressing cells (Figure 5A). A significant increase in TPT accumulation was observed for Cremophor^®^ EL in ADR cells (1.7-fold, *p* < 0.05) and for Tween^®^ 80 in MX100 cells (1.5-fold, *p* < 0.01) only. When TPT in the modified Taxol^®^ formulation was orally administered to rats, C_max_ (approximately 3.4-fold, *p <* 0.01) and AUC_INF_ (approximately 3.9-fold, *p <* 0.01) of TPT were remarkably enhanced, and V_d_/F and Cl_t_/F of the drug were substantially diminished to 27% (*p <* 0.01) and 24.9% (*p <* 0.01), respectively, as compared to those observed with TPT dissolved in saline (Figure 5B and Table 2). The improvement of oral TPT BA by this formulation (Table 2) was approximately 1.9~2.7-fold higher than that by the dual inhibitors (Table 1).

## 4. Discussion

Our laboratory’s research is focused on the development of more effective and less toxic modulators of efflux transporters to overcome the limitations of third-generation P-gp inhibitors, which have failed in the clinic [14,16]. Our previous studies concentrated on the search for novel P-gp inhibitors derived from various natural sources and on the understanding of their in vitro and in vivo effects on the PK profiles of potential anticancer drugs (P-gp substrates) [27,28,29,30,31,32] as therapeutic alternatives to third-generation P-gp inhibitors. In the present study, we investigated the potential for fourth-generation inhibition by studying compounds derived from natural sources/derivatives that were dual inhibitors targeting both P-gp and BCRP. Therefore, benzoxanthone derivatives (**2** and **3**) [30], coumarin derivatives (**LL344** and **LL347**) [27], and a modified xanthone derivative (**13-1**) were selected from the compounds showing P-gp inhibitory activity in DNR (P-gp substrate) accumulation and efflux studies, as well as ATPase activity analysis. All the selected derivatives increased DNR accumulation in the resistant cells by over two-fold compared to DNR-only treatment [27,30] (Figure 2A). In addition, we used low working concentrations of the compounds in vitro (Table S1) and in vivo (2 mg/kg) to minimize their toxicity in the present study.

The BCRP inhibitory activity of these selected P-gp inhibitors was monitored in TPT accumulation and TPT-induced cytotoxicity experiments. The in vitro results showed that the derivatives were more effective as BCRP inhibitors than as P-gp inhibitors (Figure 2B,C). However, TPT, a substrate of P-gp and BCRP, is known to possess higher affinity for BCRP [23,37], which was confirmed by using zosuquidar and Ko143, inhibitors specific for P-gp and BCRP, respectively (Figure 1B). The BCRP-dependent pattern of TPT was also confirmed by long-term treatment with various inhibitors targeting P-gp or/and BCRP in P-gp and BCRP overexpressing cells (Figure S4A). In contrast, the results of cancer cell death induced by other P-gp/BCRP substrate drugs (MX, DNR) were different from the data of TPT (Figure S4B,C). Therefore, five of the derivatives were selected for in vivo investigation as P-gp/BCRP dual inhibitors. Of note, these five selected derivatives seemed to modulate the function of both transporters without changing their protein levels (Figure 2D).

Among the dual inhibitor candidates, compounds derived from benzoxanthone (**2**, 205.7 ± 86.7% and **3**, RB: 168.2 ± 70.5%), coumarin (**LL344**, RB: 161.6 ± 85.2%), and xanthone (**13-1**, RB: 207.2 ± 99.4%) showed a greater enhancement of oral TPT BA (Table 1) and multiple-dose TPT-induced tumor growth reduction (Figure 4A,B). This tumor growth reduction by the dual inhibitors was considered the result of the increased oral TPT BA (Table 1), although species-related differences (mouse vs. rat) could also play a role.

The enhancement in TPT BA and AUC values after co-administration with the dual inhibitor candidates (2 mg/kg) can be explained by alterations in elimination rather than in absorption, based on the reduction in Cl_t_/F with no significant difference in C_max_ values. Interestingly, the modified Taxol^®^ formulation significantly enhanced TPT absorption and decreased TPT elimination (Table 2). A moderate decrease in Cl_t_/F of TPT induced by the dual inhibitor candidates to 54.3~72.9% was also observed when the P-gp specific inhibitor (zosuquidar, 25 mg/kg [37]) and the BCRP-specific inhibitor (Ko143, 10 mg/kg [37]) were co-administered with TPT (57.4% and 45.4%, respectively) (Table S2). Meanwhile, the BCRP-specific inhibitor caused not only a delay in elimination, but also enhanced absorption (>two-fold) (Table S2). In our previous study, silymarin (10 mg/kg), a P-gp inhibitor, simultaneously decreased the V_d_/F and Cl_t_/F of PTX and increased the C_max_ value of PTX [38].

TPT elimination is mainly preceded by the hydrolysis of its lactone ring, converting to a ring-opened carboxylate form at physiological pH [22]. However, we did not take the pH change induced by a single and low dose of the dual inhibitor candidates (2 mg/kg) into consideration. Thus, we postulate that any alteration in TPT metabolism by the dual inhibitor candidates could be the reason for the reduced Cl_t_/F. Initially, we speculated that CYP3A4 enzyme was involved in TPT metabolism, as: (1) the TPT metabolite (*N*-desmethyl form) has been reported to be the product of CYP3A metabolism [22]; (2) the enzyme is mainly present in the GI tract [39] as well as the hepatic tissue [40]; and (3) a single and low dose of the dual inhibitor candidates may not induce a severe impairment of liver or kidney function in rats. If the dual inhibitor candidates increased CYP3A4 activity in the GI tract, this could have increased TPT metabolism when TPT was absorbed through the GI tract. However, no relationship was observed between CYP3A4 activity and the dual inhibitor candidates with respect to TPT metabolism (Figure S5). According to GlaxoSmithKline, TPT does not alter the in vitro activity of CYP1A2, CYP2A6, CYP2C8/9, CYP2C19, CYP2D6, CYP2E, CYP3A, or CYP4A [41]. CYP3A4 may not be involved in the decrease in Cl_t_/F of TPT observed in the present study. This implies that there may be another metabolic pathway that is inhibited by these dual inhibitor candidates, or another unidentified reason responsible for the BCRP inhibition-dependent decrease in oral TPT clearance.

Furthermore, to our knowledge, this is the first report of dual target inhibition of anticancer drug efflux transporters by formulation excipients. We developed the formulation with several excipients reported to have inhibitory activity toward P-gp or BCRP. The inhibitory effects of Cremophor^®^ EL on P-gp or/and BCRP function are well known, and the effects of Tween^®^ 80 on P-gp, but not BCRP, have been well established [19]. In order to examine the effect of excipients on P-gp/BCRP dual inhibition, we developed the TPT formulation based on the Taxol^®^ formulation with the addition of Tween^®^ 80 and PEG 400 and the replacement of ethanol with saline for in vivo study. TPT dissolved very well in the developed formulation. We confirmed the inhibitory effects of these two surfactants on both efflux transporters in our study (Figure 5A). In particular, Tween^®^ 80 was found to be an efficient inhibitor of BCRP function, as evidenced by the increased TPT accumulation in BCRP-overexpressing cells (Figure 5A). In vivo, Tween^®^ 80 (1–2.5%) improved oral TPT BA (over 1.5-fold), with an increase in C_max_ and AUC_INF_ and a significant decrease in V_d_/F (Table S3). When all excipients were mixed properly, the modified Taxol^®^ formulation (22.5% Cremophor^®^ EL, 4.5% PEG 400, 0.45% Tween^®^ 80, 62.55% saline, and 10% DMSO) played an important role in the enhancement of oral TPT BA (approximately four-fold), with a significant increase in drug absorption and exposure in the body (Figure 5B and Table 2). Excipients in the formulation showed stronger effects on TPT absorption by inhibiting P-gp and BCRP function in the GI tract [6] when the anticancer drug passed through the tract (T_max_ ~1 h in Table 2) and on TPT clearance by dual inhibition in the elimination organs [6]. DMSO, which was used for dissolving the dual inhibitor candidates and Ko143, has not been reported to have any effect on the target efflux transporters. The TPT formulation composed with the surfactants which inhibited P-gp/BCRP function increased oral TPT BA much higher than the dual transporter inhibitors (compounds) in the present study. This highlights the promising drug formulation-based strategy to overcome MDR in chemotherapy.

## 5. Conclusions

In this study, the successful enhancement of oral TPT BA by P-gp/BCRP dual inhibition was demonstrated. The results showed the enhanced formulation-induced TPT absorption, and the retarded formulation- and dual inhibitor-mediated TPT elimination. P-gp inhibitors, derived from natural compounds and reported in our previous publications, were confirmed as dual inhibitors targeting BCRP as well. The dual inhibitors increased TPT accumulation in resistant cells overexpressing P-gp or BCRP, and improved oral TPT BA in rats, showing greater inhibition of BCRP. The dual inhibitors significantly enhanced tumor growth inhibition following the administration of a low dose of oral TPT in xenograft mice. Moreover, the modified Taxol^®^ formulation (22.5% Cremophor^®^ EL, 4.5% PEG 400, 0.45% Tween^®^ 80, 62.55% saline, and 10% DMSO) also significantly enhanced oral TPT BA. The combination of the dual-target inhibition of anticancer drug efflux transporters, natural compound derivatives, and drug formulation can used to overcome the limitations of third generation P-gp inhibitors.

## Figures and Tables

**Table 1:** Pharmacokinetic parameters of TPT following oral administration with or without P-gp and BCRP dual inhibitor candidates.

PK Parameters	Oral Administration					
	TPT	#2 + TPT	#3 + TPT	LL344 + TPT	LL347 + TPT	#13-1 + TPT
C_max_ (μg/mL)	0.727 ± 0.265	1.14 ± 0.215	1.02 ± 0.266	0.832 ± 0.266	0.604 ± 0.189	0.938 ± 0.375
T_max_ (h)	(0.5–1.0))	(0.5–1.0)	(0.5–1.0)	(0.5–1.0)	(0.5–2.0)	(0.25–2.0)
AUC_INF_ (μg·h/mL)	2.36 ± 0.543	4.86 ± 2.02	3.97 ± 1.71	3.82 ± 2.01	3.33 ± 0.365	4.90 ± 2.63
t_1/2_ (h)	3.39 ± 1.09	4.88 ± 2.70	4.06 ± 2.41	4.40 ± 1.91	5.95 ± 1.63	6.80 ± 5.61
V_d_/F (L)	42.1 ± 14.1	29.9 ± 14.8	27.2 ± 8.93	37.2 ± 16.6	51.1 ± 11.1	41.3 ± 30.3
Cl_t_/F (L/h)	8.94 ± 2.58	4.85 ± 2.22	6.52 ± 4.71	6.34 ± 3.01	6.06 ± 0.671	5.14 ± 2.87
RB (%)	100.0	205.7 ± 86.7	168.2 ± 70.5	161.6 ± 85.2	140.8 ± 15.4	207.2 ± 99.4

**#2**: compound 2; **#3**: compound 3; **LL344**: compound LL344; **LL347**: compound LL347; **#13-1**: compound 13-1.

**Table 2:** Pharmacokinetic parameters obtained following the oral administration of different formulations of TPT to rats.

PK Parameters	Oral Administration	
	TPT in Saline	TPT in Modified Taxol^®^ Formulation ^1^
C_max_ (μg/mL)	0.214 ± 0.141	0.727 ± 0.265 **
T_max_ (h)	(0.05–0.5)	(0.5–1.0)
AUC_INF_ (μg·h/mL)	0.611 ± 0.209	2.36 ± 0.543 **
t_1/2_ (h)	3.06 ± 1.34	3.39 ± 1.09
V_d_/F (L)	155.2 ± 79.9	42.1 ± 14.1 **
Cl_t_/F (L/h)	35.9 ± 11.4	8.94 ± 2.58 **
RB (%)	100.0	387.0 ± 88.9

** *p* < 0.01. ^1^ 22.5% Cremophor^®^ EL; 4.5% PEG 400; 0.45% Tween^®^ 80; 62.55% isotonic saline solution; 10% DMSO.

## Data Availability

Data are available in a publicly accessible repository.

## Supplementary Materials

The following are available online at https://www.mdpi.com/article/10.3390/pharmaceutics13040559/s1, Figures S1‒S5: The structure of P-gp inhibitors; P-gp or/and BCRP substrate anticancer drug accumulation analysis; In vitro study in human colon cancer cell, HT29; The effects of different anticancer drugs on cancer cell death; CYP3A4 activation assay with P-gp and BCRP dual inhibitor candidates in vitro. Tables S1‒S3: Working concentration of dual inhibitors in vitro; Effects of P-gp and/or BCRP inhibitor on PK parameters of TPT following oral administration in rats; Effects of Tween 80 on PK parameters of TPT following oral administration in rats.
