# Supplementary material for: Dual Inhibition of P-gp and BCRP Improves Oral Topotecan Bioavailability in Rodents
Source: Pharmaceutics. 2021 Apr 15;13(4):559. doi: 10.3390/pharmaceutics13040559 (PMC8071537; doi:10.3390/pharmaceutics13040559)
Supplement: Supplementary file 1 [file pharmaceutics-13-00559-s001.zip › pharmaceutics-1161002-supplementary.pdf]

pharmaceutics

Supplementary Materials: Dual Inhibition of P-gp and BCRP Improves Oral Topotecan Bioavailability in Rodents

Jaeok Lee, Jiyeon Kang, Na Yun Kwon, Aneesh Sivaraman, Ravi Naik, So Young Jin, A Reum Oh, Jae-Ho Shin, Younghwa Na, Kyeong Lee and Hwa Jeong Lee

1. Materials

Verapamil (VER) and elacridar (ELA) were purchased from Mesochem (Beijing Mesochem Technology Co., Ltd., China). The CYP3A4 activity assay kit was purchased from BioVision (BioVision Incorporated, USA).

2. Cell Survival Study

MCF-7/ADR and MCF-7/MX100 cells (9×10^3 in 96-well plates) and HT29 cells (6×10^3 in 96-well plates) were incubated with TPT with or without VER (a P-gp inhibitor), zosquidar, Ko143, and ELA (a P-gp and BCRP inhibitor) for 48 h. MX and DNR were treated with or without the inhibitor candidates for 2 h and then incubated in normal fresh medium for another 70 h. At the final time point, the incubation was stopped, and sulforhodamine B staining was performed. The absorbance was measured at 515 nm (Multiskan GO with Cuvette function #1510-04234, Thermo Fisher Scientific, Finland). The assay was performed in triplicate.

3. Substrate Drug Accumulation Study by Flow Cytometry

The cells (1×10^6–3×10^6) were incubated in pre-warmed phenol red-free media (500 μL) with 10 μM DNR in the presence and/or absence of 1 μM zosuquidar for 30 min or 200 μM TPT for 30–120 min at 37 °C. TPT accumulation in cells resuspended in cold PBS was analyzed by flow cytometry (NovoCyte 2060R, ACEA Biosciences Inc., USA). The assay was performed using at least three different biological duplicates.

4. Pharmacokinetic Studies

4.1. Drug Formulations

In the modified Taxol® formulation, TPT was prepared at a concentration of 2 mg/mL immediately prior to use. TPT was also dissolved in isotonic saline at a concentration of 2.5 mg/mL. Zosquidar and Ko143 were dissolved in DMSO at 2.5 mg/mL and 1.0 mg/mL, respectively, and each compound in DMSO was mixed with TPT (2 mg/mL) in the DMSO-free modified Taxol® formulation for oral administration. Tween® 80 was dissolved in TPT/saline to obtain final concentrations of 1% and 2.5%.

4.2. Oral Administration and Plasma Sampling

For PK studies, healthy SD rats (male, 7–8-weeks old, 224–289 g) (n = 4–6) were used for the oral administration of TPT (20 mg/kg) with or without zosuquidar (25 mg/kg), Ko143 (10 mg/kg), and the combination of zosuquidar and Ko143 in the modified Taxol® formulation. After drug administration, blood sampling was performed at 0, 0.05, 0.12, 0.25, 0.5, 1, 2, 3, 4, 6, 10, and 24 h.

The SD rats (male, 7–8-weeks old, 235–265 g) were divided into four groups (n = 6–8): TPT intravenous (IV) control (4 mg/kg), TPT PO control (40 mg/kg), TPT PO with 1% Tween® 80, and TPT PO with 2.5% Tween® 80. Blood sampling was done at 0, 0.033, 0.083, 0.25, 0.5, 1, 2, 3, 5, 7, 9, and 24 h after a single IV injection and at 0, 0.033, 0.083, 0.25, 0.5, 1, 2, 3, 5, 7, 9, 12, and 24 h after a single PO administration.

Citation: Lee, J.; Kang, J.; Kwon, N.Y.; Sivaraman, A.; Naik, R.; Jin, S.Y.; Oh, A.R.; Shin, J.-H.; Na, Y.; Lee, K.; et al. Dual Inhibition of P-gp and BCRP Improves Oral Topotecan Bioavailability in Rodents. *Pharmaceutics* **2021**, *13*, x. https://doi.org/10.3390/pharmaceutics13040559

Academic Editor: Yonghyun Lee

Received: 12 March 2021
Accepted: 13 April 2021
Published: 15 April 2021

Publisher's Note: MDPI stays neutral with regard to jurisdictional claims in published maps and institutional affiliations.

Copyright: © 2021 by the authors. Submitted for possible open access publication under the terms and conditions of the Creative Commons Attribution (CC BY) license (http://creativecommons.org/licenses/by/4.0/).

4.3. PK Analysis

Pheonix WinNonlin® software (version 8.0–8.1, Pharsight Corporation, Mountain View, CA, USA) was used to estimate the PK parameters following oral TPT administration in rats. Non-compartmental analysis was performed using the plasma TPT concentration–time profiles to obtain the following PK parameters: initial plasma concentration (C_0), area under the plasma concentration–time curve from zero to infinity (AUC_{INF}), elimination half-life ($t_{1/2}$), apparent volume of distribution (V_d) after oral administration (V_d/F), total clearance (Cl_t), and total oral clearance (Cl_t/F). The maximum plasma concentration (C_{max}) and the time required to reach C_{max} (T_{max}) were directly determined from the plasma TPT concentration–time curve. The absolute BA (AB) and the relative BA (RB) of TPT were calculated using the Equations 1 and 2:

$$AB\ (\%) = (AUC_{INF}\ \text{PO administration})/(AUC_{INF}\ \text{IV control}) \times 100 \quad (1)$$

$$RB\ (\%) = (AUC_{INF}\ \text{PO co-administration})/(AUC_{INF}\ \text{PO control}) \times 100 \quad (2)$$

5. CYP3A4 Activity Assay

The effects of P-gp/BCRP dual inhibitor candidates on CYP3A4 enzyme activity were studied by using a fluorometric CYP3A4 activity assay kit according to the manufacturer's instructions. Human CYP3A4 converted the non-fluorescent CYP3A4 substrate to a fluorescent metabolite (Em/Ex = 535/587 nm) in the buffer containing an NADPH-generating system and β-$NADP^+$. The vehicle (0.3% DMSO) was used as the control for the dual inhibitor candidates and the negative control comprised the reaction mixture without CYP3A4. Ketoconazole (30 μM) was used as the enzyme inhibitor control, and resorufin was used to construct the standard curve. Each dual inhibitor candidate was added at a final concentration of 30 μM. The reaction was monitored in kinetic mode for 35 min using a microplate multireader (Infinite F200 PRO, Tecan, Switzerland).

6. Statistical Analysis

Tukey's HSD or Dunnett's T3 test in conjunction with one-way ANOVA (Free GraphPad Prism, version 8.3.0, La Jolla, USA) was used for the statistical analysis of DNR accumulation, cell survival, and PK analysis. TPT accumulation was analyzed with Student's *t*-test/F-test. PK parameters are presented as mean ± S.D., and other data are presented as mean ± S.E.M. Statistical significance was considered to be p values < 0.05.

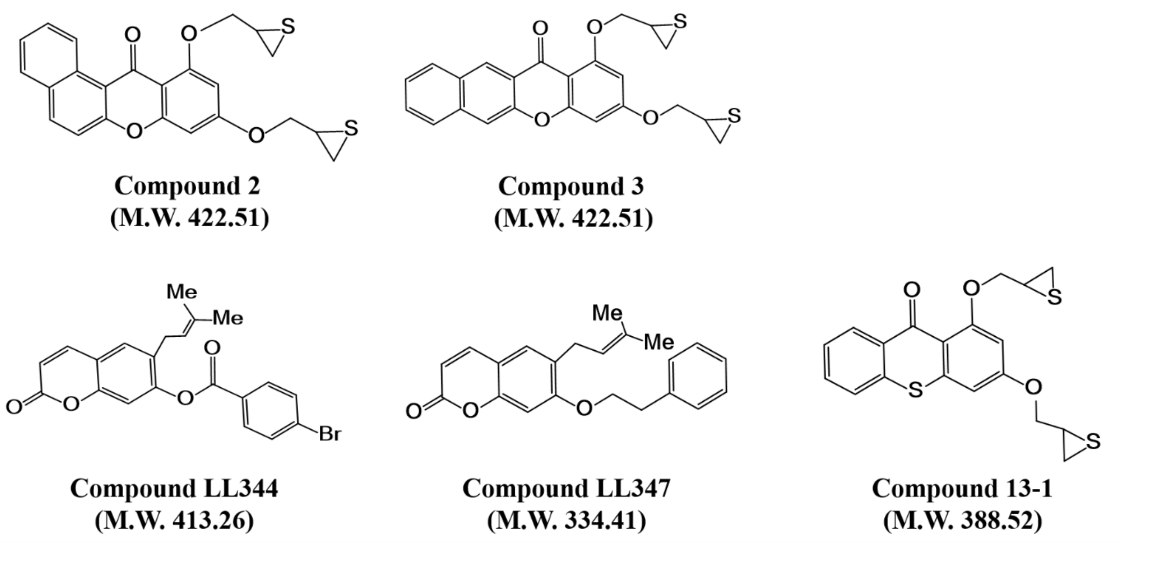

Figure S1. The structure of P-gp inhibitors. Benzoxanthone analogues, #**2** and #**3**; coumarin derivatives, **LL344** and **LL347**; xanthone derivative #**13-1**. M.W., molecular weight.

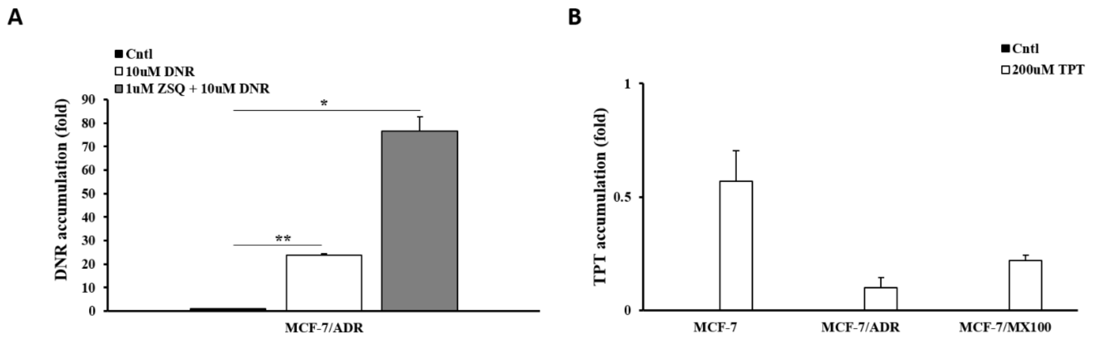

Figure S2. P-gp or/and BCRP substrate anticancer drug accumulation analysis. (**A**) DNR accumulation in P-gp over-expressed cells. (**B**) TPT accumulation. *, $p < 0.05$; **, $p < 0.01$.

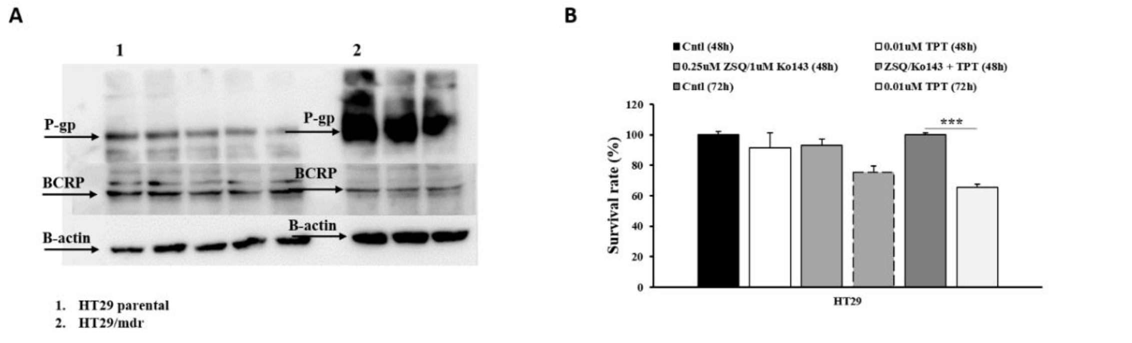

Figure S3. In vitro study in human colon cancer cell, HT29. (**A**) Endogenous P-gp and BCRP proteins expression in HT29 cells. Lane 1, HT29 cells and lane 2, HT29/mdr cells overexpressed P-gp by colchicine treatment. (**B**) Survival rate of TPT with specific P-gp and BCRP dual inhibitors. Cntl, control; ZSQ, zosuquidar. ***, $p < 0.001$.

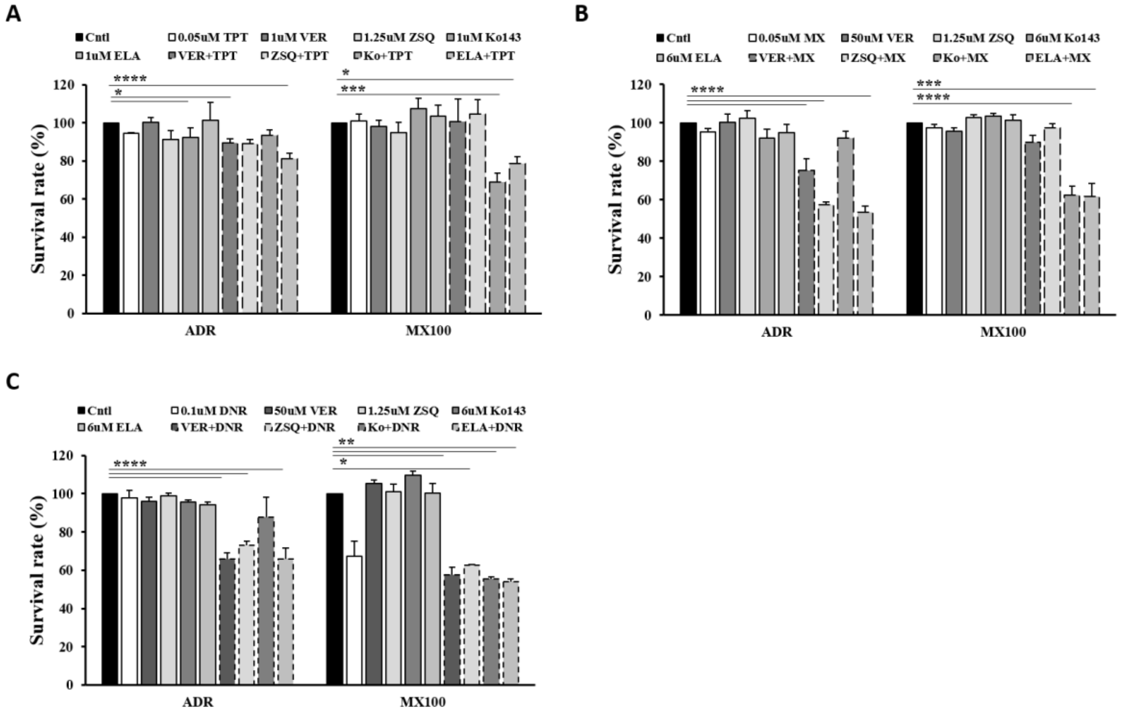

Figure S4. The effects of different anticancer drugs, P-gp- or/and BCRP-dependent, on cancer cell death. (**A**) TPT treatment with P-gp or/and BCRP inhibitors for 48 h. (**B**) and (**C**)-MX and DNR treatment with P-gp or/and BCRP inhibitors for 2 h (total incubation for 72 h). Cntl, control; VER, verapamil (P-gp inhibitor); ZSQ, zosuquidar (specific P-gp inhibitor); Ko, Ko143 (specific BCRP inhibitor); ELA, elacridar (P-gp/BCRP inhibitor). *, $p < 0.05$; **, $p < 0.01$; ***, $p < 0.001$; ****, $p < 0.0001$.

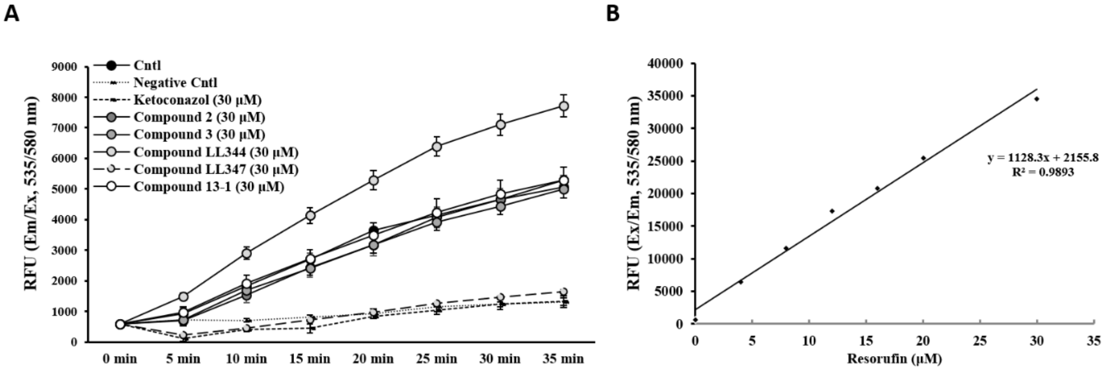

Figure S5. CYP3A4 activation assay with P-gp and BCRP dual inhibitors in vitro. (**A**) CYP3A4 assay with dual inhibitors and (**B**) Resorufin standard curve. Bars represent S.E.M. (n = 3-4). Cntl, solvent control; Ketoconazol, inhibitor control.

Table S1. Working concentration of dual inhibitors in vitro.

Incubation time		#2	#3	LL344	LL347	#13-1
30 min	Concentration (μM)	50.00	12.50	50.00	12.50	12.50
	Survival rate (%)	94.38	92.40	96.82	96.25	79.45
48 h	Concentration (μM)	6.25	6.25	50.00	6.25	3.125
	Survival rate (%)	96.80	96.00	97.65	85.18	92.39

#**2**: compound 2; #**3**: compound 3; **LL344**: compound LL344; **LL347**: compound LL347; #**13-1**: compound 13-1.

Table S2. Effects of P-gp and/or BCRP inhibitor on PK parameters of TPT following oral administration to rats.

PK Parameters	Oral Administration			
	**TPT/Modified Taxol® Formulation**	**ZSQ (25 mg/kg) +TPT**	**Ko143 (10 mg/kg) + TPT**	**ZSQ/Ko + TPT**
C_{max} (μg/mL)	0.727 ± 0.265	0.987 ± 0.422	1.60 ± 0.318**	2.40 ± 0.813
T_{max} (h)	(0.5–1.0)	(1.0)	(0.5–2.0)	(0.5–1.0)
AUC_{INF} (μg·h/mL)	2.36 ± 0.543	4.20 ± 1.14	5.67 ± 2.33	7.71 ± 1.67 *
$t_{1/2}$ (h)	3.39 ± 1.09	5.00 ± 0.908	2.72 ± 1.14	2.98 ± 1.48
V_d/F (L)	42.1 ± 14.1	38.3 ± 19.7	15.6 ± 8.58*	11.5 ± 5.88 *
Cl_t/F (L/h)	8.94 ± 2.58	5.13 ± 1.83	4.06 ± 1.78*	2.70 ± 0.678 **
**RB (%)**	**100.0**	**177.6 ± 48.4**	**240.0 ± 98.6**	**326.1 ± 70.6**

ZSQ: zosuquidar; ZSQ/Ko: the mixture of zosuquidar and Ko143. *n* = 4–6 per group. * $p < 0.05$; ** $p < 0.01$.

Table S3. Effects of Tween 80 on PK parameters of TPT following oral administration to rats.

PK Parameters	IV (4 mg/kg)	PO (40 mg/kg)		
	**TPT/Saline**	**TPT/Saline**	**+1.0% Tween 80**	**+2.5% Tween 80**
C_0 (μg/mL)	2.64 ± 0.764	-	-	-
C_{max} (μg/mL)	-	0.235 ± 0.158	0.639 ± 0.384	0.711 ± 0.294**
T_{max} (h)	-	(0.033–0.5)	(0.033–1.0)	(0.033–0.5)
AUC_{INF} (μg·h/mL)	1.37 ± 0.280	2.30 ± 0.706	3.99 ± 1.78	3.42 ± 0.687*
$t_{1/2}$ (h)	0.914 ± 0.115	16.5 ± 5.26	12.4 ± 5.35	12.0 ± 5.02
V_d (L)	1.01 ± 0.233	-	-	-
V_d/F (L)	-	109.2 ± 33.20	48.4 ± 14.0*	50.3 ± 17.1*
Cl_t (L/h)	0.768 ± 0.171	-	-	-
Cl_t/F (L/h)	-	4.84 ± 1.59	3.01 ± 1.22	3.07 ± 0.690
AB (%)	-	16.8 ± 5.16	29.2 ± 13.0	25.0 ± 5.02
RB (%)	-	100.0	173.9 ± 77.3	149.0 ± 29.9

n = 6–8 per group. * $p < 0.05$; ** $p < 0.01$.
